# Effect of Long-Term Xanthophyll and Anthocyanin Supplementation on Lutein and Zeaxanthin Serum Concentrations and Macular Pigment Optical Density in Postmenopausal Women

**DOI:** 10.3390/nu10080959

**Published:** 2018-07-25

**Authors:** Begoña Olmedilla-Alonso, Rocío Estévez-Santiago, José-Manuel Silván, Milagros Sánchez-Prieto, Sonia de Pascual-Teresa

**Affiliations:** Instituto de Ciencia y Tecnología de Alimentos y Nutrición (ICTAN-CSIC), C/José Antonio Novais, 10, 28040 Madrid, Spain; rocioestevez4@gmail.com (R.E.-S.); jm.silvan@csic.es (J.-M.S.); msprieto@ictan.csic.es (M.S.-P.); s.depascualteresa@csic.es (S.d.-P.-T.)

**Keywords:** lutein, zeaxanthin, xanthophylls, carotenoids, anthocyanins, macular pigment

## Abstract

Xanthophylls (lutein, L; zeaxanthin, Z) and anthocyanins are often included in food supplements to improve ocular health. There are no dietary reference intakes for them. The aim was to assess the effects of L, Z and anthocyanin supplementation on short and long-term lutein status markers (serum concentration and macular pigment optical density (MPOD)). Seventy-two postmenopausal women were randomized into a parallel study of 8 months: Group A—anthocyanines (60 mg/day); Group X—xanthophylls (6 mg L + 2 mg Z/day); Group X+A—anthocyanines (60 mg/day) + xanthophylls (6 mg L + 2 mg Z/day). At the beginning of the study, 4 and 8 month serum L and Z concentrations were determined (HPLC), as well as L, Z and anthocyanine dietary intake and MPOD (heterochromic flicker photometry). Baseline concentrations of L (0.35 ± 0.19 μmol/L), Z (0.11 ± 0.05 μmol/L), L+Z/cholesterol/triglycerides (0.07 ± 0.04 μmol/mmol) increased in Group X (2.8- and 1.6-fold in L and Z concentrations) and in group XA (2- and 1.4-fold in L and Z concentrations). MPOD (baseline: 0.32 ± 0.13 du) was not modified in any of the groups at the end of the study. There were no differences in the dietary intake of L+Z and anthocyanin at any point in time in any group. Supplementation of L and Z at a dietary level provoked an increase in their serum concentration that was not modified by simultaneous supplementation with anthocyanins.

## 1. Introduction

Xanthophylls and anthocyanins are plant pigments that are consumed as part of a healthy diet. These two groups of phytochemicals have shown to be bioactive in different cardiovascular and retina models and to produce beneficial effects in epidemiological and clinical studies. Their protective effects involve different mechanisms, some of them shared by both groups of compounds, e.g., antioxidant activity and inflammatory modulation [1,2,3,4,5].

Lutein and zeaxanthin are the dietary xanthophylls that constitute the macular pigment (MP) and act by filtering blue light, and as antioxidants, whereby they protect the retina from oxidative damage induced by light and a high rate of oxidative metabolism in this tissue [6]. The MPOD is positively associated with dietary and serum lutein and zeaxanthin concentrations [7,8,9,10,11]. Lutein supplementation, using different protocols, doses (including doses at nutritionally achievable levels) and sources of lutein (diet and capsules) has shown its effectivity in increasing serum concentrations and can increase the macular pigment optical density (MPOD) in most, but not all, healthy subjects [7,12]. The increment in the MP density has been related to improved visual function [13,14,15] and brain age-related diseases [16] and with a decreased risk of progression of intermediate age-related macular degeneration (AMD) to late AMD [2,17] that is a major cause of blindness in the elderly population in the developed world [18]. High lutein and zeaxanthin intakes are associated with a lower risk of AMD, a disease for which oxidative, vascular and genetic etiological hypotheses have been proposed.

Among the major contributors to lutein and zeaxanthin dietary intake (fruits, vegetables and eggs), fruit intake shows a higher correlation with MPOD than vegetables intake [19], and the correlations between the amounts of lutein and zeaxanthin provided by fruits (µg/day) is higher that that of vegetables [20]. A lower risk of AMD was associated with a higher intake of fruits but not of vegetables in an epidemiological study assessing MPOD and fruit and vegetable intake [20,21]. As the contribution of fruits to the lutein and zeaxanthin dietary intake is lower than that of vegetables (nearly 20-fold less in subjects aged 45 to 65 years) [20], its higher correlation with MPOD could be partially explained by the higher bioavailability of lutein and zeaxanthin from fruits [22] or because of the presence of other micronutrients and bioactive compounds present in fruits (e.g., fiber and polyunsaturated fatty acid intake are also directly related to MPOD) [7,10].

To date, there are no dietary reference intakes for these bioactive components, although lutein meets a set of criteria in relation to the promotion of optimal health and/or the prevention of chronic diseases to be considered for dietary reference intakes—like recommendations [23,24]. However, xanthophylls and anthocyanins are often included, together or independently, in commercially available food supplements to improve ocular health. On the other hand, there are few studies in the scientific literature that have shown an effect of their combined consumption, and the few studies available are not conclusive [25,26,27]. Therefore, there is still a need for intervention studies in well-defined population groups, mainly in those who could benefit from a higher intake of lutein, e.g., older subjects, as MPOD decreases with age [19], which increases the risk of age-related ocular diseases. These studies will allow an increase in the knowledge of the dose–effect relationship and the interactions between these two groups of food components, thereby strengthening the scientific basis for public health advice in this area. Thus, the aim of this parallel study was to assess the effect of a dietary supplementation with xanthophylls and anthocyanins on short and long-term lutein status markers (serum concentration and MPOD) in postmenopausal women.

## 2. Materials and Methods

### 2.1. Subjects and Study Design

Seventy-five women were enrolled and randomized into a parallel study of 8 months duration, and 72 completed the study. The inclusion criteria were as follows: age (50–70 years), amenorrhea (>2 years) and body mass index (BMI) (25–33 kg/m^2^). Participants were selected from 90 subjects who were interested and contacted through advertisements in research centers, universities and several noticeboards (12 did not meet the inclusion criteria and 3 decided not to participate). The exclusion criteria were cholesterolemia (>6.2 mmol/L), the use of drugs or foods to control cholesterol levels, the use of restrictive diets and the use of hormone replacement therapy or chronic diseases that can affect any of the variables analyzed. Participants were randomized into three groups: Group A—anthocyanines (60 mg/day); Group X—xanthophylls (6 mg lutein + 2 mg zeaxanthin/day); Group X+A—anthocyanines (60 mg/day) + xanthophylls (6 mg lutein + 2 mg zeaxanthin/day). Supplements were prepared by HC Clover Ps (Madrid, Spain).

Blood samples were collected at the start of the study and after 4 and 8 months for the analysis of serum lutein and zeaxanthin (by HPLC) and the lipid profile (cholesterol and triglycerides (TG)). The macular pigment optical density (MPOD) was determined using heterochromic flicker photometry (MPS 9000 desktop device, Macular Pigment Screener Elektron PLC, Cambridge, UK).

The study was approved by the Ethics Committee for Clinical Research of the Hospital Universitario Puerta de Hierro-Majadahonda (Madrid, Spain) (Acta no 283, dated 17 December 2012) and the Bioethics Committee of the Spanish National Research Council (CSIC) (dated 30 May 2014). All subjects gave their written informed consent after receiving oral and written information about the study.

### 2.2. Lutein, Zeaxanthin and Lipid Analysis in Blood

Lutein and zeaxanthin levels were determined by high performance liquid chromatography (HPLC) using a system consisting of a model 600 pump, a Rheodyne injector, a 2998 photodiode array (PDA) detector (Waters, Milford, MA, USA) and a C30 YMC column (5 μm, 250 × 4.6 mm i.d.) (Waters, Wilmington, MA, USA) with a guard column (Aquapore ODS type RP-18). Mobile phase: methanol with 0.1% trimethylamine:metihyl-tert-butyl-ether (MTBE) in a linear gradient from 95:5 to 70:30 in 30 min, to 50:50 in 20 min, and this proportion was maintained for 35 min. Flow rate: 1 mL/min. The detection was performed at a wavelength of 450 nm. Chromatograms were processed using Empower 2 software (Waters, Milford, MA, USA). Carotenoid identification was done by comparing the retention times with those of authentic standards and online UV-VIS spectra [28].

Carotenoid extraction from serum samples was performed using a procedure described elsewhere with a slight modification [29]. Briefly, 800 μL of serum was added to 800 μL of ethanol, which was vortexed and extracted twice with 800 μL of hexane: dichloromethane (5:1) and stabilized with 0.1 g/L butylated hydroxyltoluene (BHT). Organic phases were pooled, evaporated to dryness under a nitrogen atmosphere and reconstituted with 250 μL of a solution of methanol:MTBE (1:1) and injected (20 μL) onto the HPLC system.

MTBE, methanol, ethanol, dichloromethane, hexane, trimethylamine, ammonium acetate, BHT and tetrahydrofuran were supplied by Panreac (Barcelona, Spain). Lutein (xanthophyll from marigold) was obtained from Sigma Chemical Co. (St. Louis, MO, USA), and zeaxanthin was purchased from Sigma Aldrich (Madrid, Spain). Standard solutions were prepared from 1 mg of lutein and of zeaxanthin dissolved in 25 mL tetrahydrofuran with 0.01% BHT in each case. The $E_{1\%}^{1cm}$ values and wavelengths used were as follows: lutein, 2550 at 445 nm; zeaxanthin, 2540 at 450 nm [28]. Working solutions were obtained from different volumes of standard solutions dissolved in methanol:MTBE (1:1). The concentrations of the carotenoids in the curve were: 0.08–1.10 μg/mL for lutein (*R*^2^ = 0.999) and 0.05–0.45 μg/mL for zeaxanthin (*R*^2^ = 0.999).

The blood total cholesterol, high-density lipoprotein (HDL) cholesterol and serum TG levels were analyzed using a dry-slide enzymatic method (Vitros 5,2 FS, Ortho Clinical Diagnostics, Rochester, NY, USA). The Friedewal equation was used to calculate the low-density lipoprotein (LDL) cholesterol concentration [30].

### 2.3. Dietary Intake Assessment

Three-day food records involving 24 h recalls were used to calculate the recent dietary intake; one day coincided with a weekend or holiday and they were carried out within a period of 7 to 10 days. For the first recall, the participants underwent a face-to-face encounter with a specialized interviewer, normally the same person who, subsequently, performed the other two recalls by telephone [19,20]. The amounts consumed were estimated in units (fruits), portions or household servings that were standardized for this study [31] and, the food intake was calculated in grams/day, which served as the basis for the determination of daily lutein and zeaxanthin intakes using a database included in a software application for the calculation of dietary intake of individual carotenoids [32]. Anthocyanidin intake was estimated as the sum of derivatives of cyanidin, delphinidin, peonidin, petunidin, pelargonidin and malvidin. Anthocyanin data were obtained from information relating to the polyphenol content in foods from the Phenol-Explorer database [33], as well as the content of eggplant [34], pinto beans [35] and pomegranate [36]. Foods included in the assessment were apricots, beans (pinto), blackberries (raw and jam), blueberries (highbush and lowbush), cherries, eggplants, grapefruits (black), lettuces (red), olives (black), pomegranates, plums (black), raspberries, strawberries and grapes (red). To evaluate the lipid and energy intake, we employed a software application that is widely used in Spain [37].

### 2.4. Macular Pigment Optical Density Assessment

As described elsewhere [19,20], macular pigment optical density was assessed using an MPS 9000 desktop device (Macular Pigment Screener, Elektron PLC, Cambridge, UK) that applies the principles of heterochromatic flicker photometry. This technique and its reliability are described by van der Veen et al. [38]. The test consists of two stages, for central and peripheral viewing, and the subjects were required to press a response button as soon as they detected flicker. The subjects started by fixating on the central stimulus, a 1 degree central target (the flicker rate was initially set to 60 Hz which then gradually reduced at a rate of 6 Hz s^−1^). The process was repeated for a series of green-blue luminance ratios. The observer then fixated on a red 2° diameter target placed 8° eccentrically and a second set of data were recorded for peripheral viewing [39]. The MPOD was measured in density units (du) and ranged from 0 to 1.

### 2.5. Statistics

A sample size calculation was performed on the basis of a mean value for MPOD of 0.40 du. A sample of 75 subjects (SD = 0.12) was found to be necessary to obtain a 10% difference in the MPOD (0.07 du) with 80% power and an alpha error of 0.05. Descriptive statistics (mean, standard deviation, 95% confidence interval (CI) and median) were calculated for all of the analytes. Statistical comparisons were carried out with nonparametric methods (Wilcoxon and Kruskall–Wallis tests) and differences according to treatment were assessed using one-way ANOVA and the post-hoc Bonferroni test. Relationships among variables in serum and the MPOD were established using Spearman’s rho correlation coefficient. Statistical significance was set at *p* < 0.05, and analyses were performed with IBM SPSS Statistics 22.0 (Armonk, NY, USA; IBM Corp.)

## 3. Results

The concentrations of lutein, zeaxanthin, lutein + zeaxanthin/cholesterol + TG, cholesterol, HDL, LDL, TG and MPOD at baseline are shown in Table 1. 

**Figure 1:**
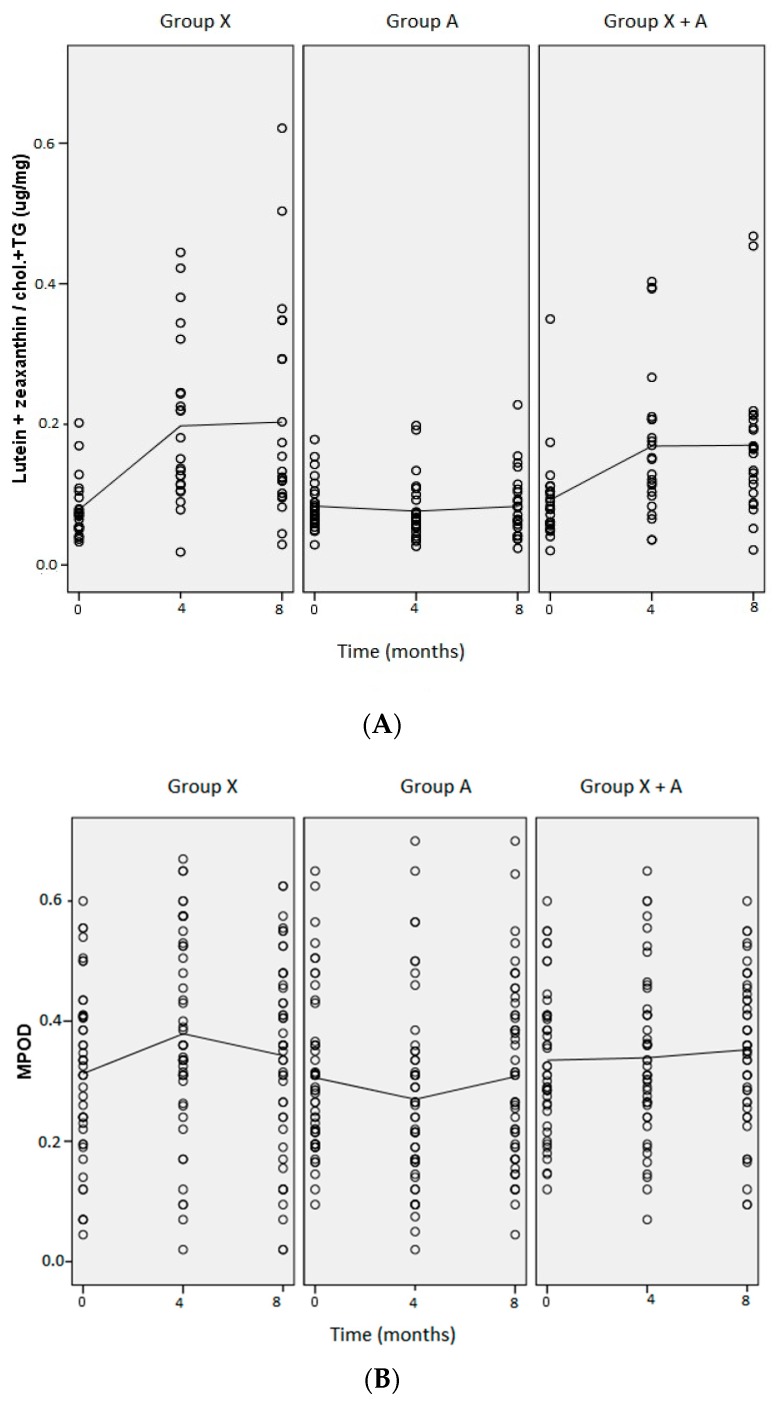
Concentrations of L+Z /chol. + TG in serum (**A**) and MPOD units (**B**) in each group of treatment at baseline, and after 4 and 8 months of the study.

The lutein, zeaxanthin and lipid concentrations in blood and MPOD in each of the three treatment groups throughout the study period are shown in Table 2. At baseline, there were no differences in the variables analyzed between treatment groups. Supplementation with xanthophylls (L and Z) provoked increases in serum lutein and zeaxanthin concentrations in Groups X (*p* = 0.000 for lutein and *p* = 0.011 for zeaxanthin, at 4 months) and X+A (*p* = 0.001 for lutein and *p* = 0.036 for zeaxanthin, at 4 months), which were higher in group X, although the difference was not statistically significant. In Group X, we observed 2.8 and 1.6-fold increases in serum lutein and zeaxanthin levels, respectively, whereas in Group X+A, the increments were 2 and 1.4-fold, respectively. In Group A, there were no variations in serum lutein and zeaxanthin concentrations during the study period. When serum concentrations were related to levels of circulating lipids (lutein + zeaxanthin/cholesterol + TG) (Figure 1A), the increase was 2.5-fold in Group X (*p* = 0.001, at 8 months) and 1.9-fold in Group X+A (*p* = 0.023, at 8 months). These levels of lutein and zeaxanthin, and lutein + zeaxanthin/cholesterol + TG in serum were reached at 4 months and were maintained throughout the intervention period (8 months) in the X and X+A groups. There were no variations in serum lutein and zeaxanthin concentrations in Group A or in the concentrations of total cholesterol, HDL, LDL or TG in any of the three groups. The MPOD value was not modified in any of the groups at the end of the study. Modifications in the MPOD were only observed at 4 months—an increase in Group X (*p* = 0.007) and a decrease in Group A (*p* = 0.024). MPOD did not increase at the end of the study in either of the groups taking lutein plus zeaxanthin (Groups X and X+A) (Figure 1B).

The dietary intakes of lutein plus zeaxanthin and anthocyanins (as the sum of cyanidin, delphinidin, peonidin, petunidin, pelargonidin and malvidin glycosides) in volunteers throughout the study period are shown in Table 3. Lutein and zeaxanthin were ingested by all volunteers. However, foods supplying anthocyanins were consumed only by 48 of the participants at the beginning of the study and by 32 and 40 volunteers at 4 and 8 months respectively. There was a significant increase in the dietary intakes of lutein and zeaxanthin by volunteers included in Groups X (from 0 to 8 months, *p* = 0.022) and A (from 0 to 4 months, *p* = 0.026; from 0 to 8 months, *p* = 0.034) and, there was a significant decrease in the dietary intake of anthocyanins, between the intake at baseline and at 4 months, in Group X (*p* = 0.038), Group A (*p* = 0.013) and Group XA (*p* = 0.005). There were no differences in the dietary intake of lutein plus zeaxanthin or of anthocyanins at any time point between treatment groups.

The concentration of zeaxanthin in serum was the only variable analyzed in serum that showed a significant correlation with MPOD at baseline (*ρ* = 0.231, *p* = 0.005). After 4 months of the study, only in Group X+A did serum concentrations of zeaxanthin (*ρ* = 0.306, *p* = 0.038) and lutein plus zeaxanthin (*ρ* = 0.296, *p* = 0.046) show significant correlations with MPOD. After 8 months, lutein (*ρ* = 0.371, *p* = 0.013), zeaxanthin (*ρ* = 0.481, *p* = 0.001) and lutein plus zeaxanthin (*ρ* = 0.378, *p* = 0.011) showed significant correlations with MPOD, only in Group X. However, no correlations were found when the mentioned concentrations were expressed in relation to cholesterol plus triglycerides. With respect to the relationship between MPOD and the lipids in which the xanthophylls are transported, correlations were obtained with cholesterol at 4 months in Group A (*ρ* = −0.328, *p* = 0.018) and in Group X+A (*ρ* = 0.298, *p* = 0.044). Correlations occurred with LDL-cholesterol at 4 months in Group X (*ρ* = 0.357, *p* = 0.015) and in Group A (*ρ* = −0.372, *p* = 0.007), and correlations occurred with HDL-cholesterol at 4 months in group X (*ρ* = −0.346, *p* = 0.018).

## 4. Discussion

In the present study, an intervention with xanthophylls and anthocyanins was done in a well-defined group of women aged 50–70 years, as these individuals are those who could benefit from food supplements designed to improve ocular health and decrease the risk of age-related ocular diseases. These supplements are commercially available in formulas with varied compounds and in diverse concentrations. The xanthophyll capsule provided 6 mg of lutein, similar to that contained in 100 g of spinach (boiled) [40] and 2 mg of zeaxanthin, similar to that contained in 100 g of artichokes or Brussel sprouts [40]. This amount of lutein (6 mg/capsule) is a standard mean concentration in food supplements, and although it is achievable by dietary means, it is higher than that reported by population-based studies or those involving groups of healthy adults [41,42,43] and lower than that supplied in the formula used in the AREDS2 study for age-related macular degeneration (10 and 2 mg of lutein and zeaxanthin, respectively) [17] and in most clinical trials, where lutein supplementation has ranged between 10 and 20 mg/day and zeaxanthin supplementation between 0.6 and 2 mg/day [44]. Xanthophyll capsules provided 8.5 times the mean intake of lutein in the Spanish population and 33 times that of zeaxanthin (0.7 and 0.06 mg/person/day, respectively) [43]. Anthocyanins may have estrogenic-antiestrogenic effects [45] and the amount of anthocyanins selected was slightly above the upper range of the dietary intake described (3–50 mg/person/day) [46,47] and within the range of the anthocyanin content in a wide range of food supplements [48].

Lutein and zeaxanthin concentrations in serum were slightly higher than those reported in Spanish subjects aged 45–65 years [19], and the serum concentration of lutein plus zeaxanthin was similar to [8] or higher than [49,50,51,52] concentrations described in other studies in women of comparable age in different countries with presumably different dietary habits. The MPOD in the present study was practically the same as that found in a previous study in a group of men and women aged 45–65 years [19] and similar to [8] or higher than [53] that reported in other studies on subjects within a similar age range who were free of retinal disease.

The increment in the serum lutein concentration observed with this xanthophyll supplementation—2.8-fold and 2-fold in groups X and X+A, respectively—is in agreement with the 5-fold increment described in a supplementation study with 15 mg/day (2.5 times more lutein/day than in the present study) for 5 months in European subjects. However, the increment in the serum zeaxanthin serum concentration obtained in the present study—1.6 and 1.4-fold in Groups X and X+A, respectively—with an extra dietary intake of zeaxanthin of 2 mg/day, is practically the same increment as that reported in the aforementioned healthy European subjects—2-fold, in which no zeaxanthin was supplied [54]. However, a higher increase, 6-fold, was reported in healthy young subjects (20–25 years) with an intake of 6 mg lutein + 1 mg zeaxanthin for three months [55]. On the contrary, in patients with age-related cataracts, a similar increment in serum lutein (2-fold), was obtained with similar supplementation of lutein, 6.5 mg lutein/day [12], and the highest serum lutein and zeaxanthin concentrations were reached at 4 months (2.7-fold and 1.5-fold from baseline, for lutein and zeaxanthin, respectively) and were maintained until the end of the study—8 months. However, the maximum concentration may have occurred before measurement, as a plateau in the serum response to lutein intake (15 mg/day) has been described after one month of supplementation and maintained for five months [54], even after 2 to 3 weeks of supplementation with different doses of lutein [56].

The increase in the serum concentrations of lutein and zeaxanthin in the group with simultaneous intake of xanthophylls and anthocyanins was lower, although not at a statistically significant level, with respect to that produced by the consumption of only xanthophylls, and this could be due to an insufficient sample size or because of a high variability in the volunteers’ responses, among other factors. However, anthocyanins are commonly consumed by people who take care of their ocular health, alone and in combination with xanthophylls; however, there is no information on the efficacy and pharmacokinetics of the anthocyanins [57]. A recent study showed that anthocyanins, especially, malvidin and its glycosides, are beneficial to human retinal pigment epithelial cells, and blueberry anthocyanins could inhibit the induction and progression of AMD [58]. In this study, long-term supplementation with xanthophylls at a dietary achievable level, although higher than the mean population intake [43], was effective in increasing and maintaining serum lutein concentrations comparable to those that lead to an increment in MPOP [55,59,60]. Nevertheless, lower concentrations of lutein and zeaxanthin (1.4 and 0.2 mg, respectively), similar to the dietary intake of most populations, also led to an increase in the MPOD. This was reported in subjects with retinal pigment abnormalities and/or drusen who consumed a lutein-enriched egg drink daily for twelve months. However, this was not found after consumption for 6 months [61] or 3 months [62]. With this lutein-enriched egg drink, there was simultaneous supply of docosahexanoic acid that facilitated the incorporation of lutein in the MP. However, with a higher amount of lutein, we did not observe an increment in the MPOD after 8 months of supplementation in any treatment group with a higher amount of lutein. An insufficient period of supplementation for women with the characteristics of those included in this study could be one of the reasons for the lack of an increment in the MPOD. However, it could also be due, among other factors, to a lower uptake of lutein and zeaxanthin from the serum into the retina, to a potential instability of lutein in the capsules supplied [63], to the quantity of fat in the meal during lutein supplement intake or its absence [64] and/or to the high inter-individual variability in the MPOD response to the supplementation [65].

The MPOD showed significant correlations with serum lutein and zeaxanthin in the groups supplemented with X and with X+A, but not in Group A, as was reported in other studies in subjects over the age of 45 years, in whom MPOD was associated with serum lutein and zeaxanthin concentrations [8,19]. No significant associations with lutein and zeaxanthin dietary intake were observed in this study, but they were detected in others with the dietary intake of zeaxanthin [19] and lutein plus zeaxanthin [8].

Not all of the women in the present study consumed anthocyanins in their diet, and the anthocyanin dietary intake showed an enormous variability between subjects, with an average intake of 15.6 ± 23.3 mg anthocyanin/person/day, lower than that reported in other populations, on average, 3–50 mg/person/day [46]. In three US cohorts, the consumption of anthocyanins was in the range of 12.5 to 15.2 mg/person/day, and they were supplied almost entirely by blueberries and strawberries [47]. However, in our study, 48, 32 or 40 out of 72 women, depending on the time point, consumed foods with no anthocyanin content. This might be explained by the fact that anthocyanins are present in a very small number of foods, in contrast to other more ubiquitous polyphenol groups, such as flavonols or, in this same study, carotenoids. Additionally, one should bear in mind that anthocyanins are preferably present in red fruits, which are seasonal, and the first visit of the participants in this study was carried out at the end of the spring. This could explain the higher consumption of anthocyanins encountered at baseline. The great variability in anthocyanin intake between subjects and time points of the intervention may have introduced a confounding effect.

## 5. Conclusions

Lutein and zeaxanthin supplements provoke an increase in the serum concentrations of these xanthophylls that was not modified by simultaneous supplementation with anthocyanins, all of them at dietary achievable levels, in a homogeneous group of postmenopausal women. This is a potential target group of this type of food supplement that is consumed in random concentrations in the case of anthocyanins, and there was no in vivo confirmation of the effect of these compounds when taken simultaneously on the macular pigment or on any aspect related to ocular health. However, this study should be repeated with a greater number of participants to check the differences observed when xanthophylls and anthocyanins are supplied jointly.

Moreover, neither the xanthopylls, nor the anthocyanins, or their combination, at the selected doses, were capable of increasing the MPOD after eight months of supplementation. Nevertheless, in spite of the lack of response of the MPOD, supplementation at nutritionally achievable levels is of great interest to avoid an imbalance in the dietary components due to interaction effects, for example, those described for the carotenoids on mortality in the NHANES III study [66]. On the other hand, there is a need for intake reference ranges for these bioactive compounds which are associated with optimal health and a lower risk of chronic diseases, as there is already an increasing consensus regarding lutein [24]. Thus, more nutritional strategies are needed to protect the cells of the retinal pigment epithelium in well-defined groups of subjects at risk for certain chronic diseases whose origin or development could be influenced by compounds of this type. 

## Figures and Tables

**Table 1:** Concentrations of the variables analyzed at baseline (*n* = 72).

	Mean ± SD	Median	CI_95%_
Lutein (µmol/L)	0.35 ± 0.19	0.31	0.31; 0.40
Zeaxanthin (µmol/L)	0.11 ± 0.05	0.10	0.10; 0.12
Lutein + zeaxanthin (µmol/L)	0.46 ± 0.23	0.39	0.41; 0.52
Lutein + zeaxanthin/chol. + TG (µmol/mmol)	0.07 ± 0.04	0.06	0.06; 0.08
Cholesterol (mmol/L)	5.59 ± 0.65	5.52	5.44; 5.72
HDL-cholesterol (mmol/L)	1.74 ± 0.65	1.71	1.66; 1.81
LDL-cholesterol (mmol/L)	3.39 ± 0.65	3.26	3.26; 3.55
Triglycerides (TG) (mmol/L)	0.97 ± 0.35	0.95	0.89; 1.06
MPOD (du) (*n* = 144 eyes)	0.32 ± 0.13	0.31	0.30; 0.34

Chol.: cholesterol; CI: confidence interval; HDL: high-density lipoprotein; LDL: low-density lipoprotein; MPOD: macular pigment optical density; SD: standard deviation; TG: triglycerides.

**Table 2:** Lutein, zeaxanthin, and lipid serum concentrations and MPOD at baseline and after 4 and 8 months of supplementation.

	Group X (Xanthophylls)	Group A (Anthocyanins)	Group A+X (Anthocyanines + Xanthophylls)
	Mean ± SD		
Lutein—baseline	0.34 ± 0.21 ^a^	0.34 ± 0.14 ^a^	0.39 ± 0.22 ^a^
4 months	0.95 ± 0.49 ^b^	0.34 ± 0.20 ^a^	0.81 ± 0.51 ^bc^
8 months	0.95 ± 0.67 ^b^	0.36 ± 0.25 ^a^	0.78 ± 0.47 ^bc^
Zeaxanthin—baseline	0.11 ± 0.05 ^a^	0.10 ± 0.03 ^a^	0.11 ± 0.08 ^a^
4 months	0.17 ± 0.86 ^b^	0.09 ± 0.05 ^a^	0.16 ± 0.13 ^bc^
8 months	0.16 ± 0.08 ^b^	0.10 ± 0.05 ^a^	0.15 ± 0.08 ^ac^
Lutein + zeaxanthin—baseline	0.44 ± 0.24 ^a^	0.44 ± 0.16 ^a^	0.50 ± 0.29 ^a^
4 months	1.12 ± 0.56 ^b^	0.43 ± 0.24 ^a^	0.97 ± 0.60 ^bc^
8 months	1.11 ± 0.75 ^b^	0.46 ± 0.29 ^a^	0.92 ± 0.54 ^bc^
Lutein + zeaxanthin/cholesterol + TG—baseline	0.08 ± 0.04 ^a^	0.08 ± 0.04 ^a^	0.09 ± 0.07 ^a^
4 months	0.20 ± 0.12 ^b^	0.08 ± 0.04 ^a^	0.17 ± 0.11 ^bc^
8 months	0.20 ± 0.15 ^b^	0.08 ± 0.05 ^a^	0.17 ± 0.11 ^bc^
Cholesterol—baseline	5.54 ± 0.62	5.47 ± 0.70	5.78 ± 0.57
4 months	5.80 ± 0.67	5.67 ± 0.67	5.85 ± 0.73
8 months	5.72 ± 0.73	5.59 ± 0.88	5.59 ± 0.93
HDL-cholesterol—baseline	1.63 ± 0.34	1.71 ± 0.34	1.84 ± 0.36
4 months	1.81 ± 0.47	1.74 ± 0.34	1.84 ± 0.39
8 months	1.76 ± 0.39	1.74 ± 0.29	1.89 ± 0.36
LDL-cholesterol—baseline	3.45 ± 0.67	3.29 ± 0.67	3.50 ± 0.57
4 months	3.50 ± 0.78	3.45 ± 0.67	3.55 ± 0.60
8 months	3.47 ± 0.78	3.42 ± 0.86	3.29 ± 0.78
TG—baseline	1.06 ± 0.35	0.95 ± 0.38	0.93 ± 0.29
4 months	1.07 ± 0.35	1.04 ± 0.38	1.03 ± 0.45
8 months	1.07 ± 0.46	0.96 ± 0.35	0.94 ± 0.41
MPOD—baseline	0.31 ± 0.14 ^a^	0.31 ± 0.13 ^a^	0.34 ± 0.12
4 months	0.38 ± 0.17 ^b^	0.27 ± 0.15 ^b^	0.34 ± 0.14
8 months	0.34 ± 0.16	0.31 ± 0.15	0.35 ± 0.13

L, Z and L+Z are expressed in µmol/L; L+Z/chol. + TG are expressed in µmol/mmol; Chol, HDL, LDL and TG are expressed in mmol/L; MPOD in density units. Different superscript letters within rows mean significant differences (*p* < 0.05).

**Table 3:** Dietary intakes of lutein and zeaxanthin and anthocyanins expressed as means ± SDs, (median) and (range).

	Lutein and Zeaxanthin (μg/day)			Anthocyanins ^1^ (mg/day)		
Basal (*n* = 72)	629.7 ± 613.8			25.3 ± 31.3		
	(412.4)			(14.0)		
	(40.8–3505.3)			(0.0–161.3)		
	**Group X**	**Group A**	**Group XA**	**Group X** (*n* = 23)	**Group A** (*n* = 26)	**Group XA** (*n* = 23)
Basal	634.0 ± 599.3 ^a^	564.4 ± 393.7 ^a^	702.5 ± 828.2 ^a^	17.7 ± 20.6 ^a^	21.7 ± 26.3 ^a^	37.0 ± 41.6 ^a^
	(412.4)	(465.9)	(348.9)	(11.0)	(11.0)	(24.8)
	(123.5–2932.3)	(142.9–1809.6)	(40.8–3505.3)	(0.0–67.5)	(0.0–73.9)	(0.0–161.3)
4 months	706.9 ± 572.8 ^a^	1015.0 ± 842.8 ^b^	1016.7 ± 793.0 ^a^	7.2 ± 12.5 ^b^	6.1 ± 14.5 ^b^	9.5 ± 13.0 ^b^
	(441.7)	(740.6)	(796.1)	(0.0)	(0.0)	(3.1)
	(71.2–2358.2)	(357.0–4028.9)	(200.6–3421.4)	(0–44.0)	(0.0–66.0)	(0.0–36.1)
8 months	1070.6 ± 770.4 ^ab^	1052.6 ± 1015.0 ^bc^	1036.5 ± 888.9 ^a^	9.3 ± 13.7 ^ab^	13.7 ± 18.8 ^ab^	17.8 ± 31.1 ^ab^
	(904.8)	(673.7)	(812.0)	(0.6)	(0.9)	(7.7)
	(233.1–3178.0)	(354.3–5023.3)	(210.3–3233.0)	(0.0–44.0)	(0.0–69.4)	(0.0–137.3)

^1^ Sum content of cyanidin, delphinidin, peonidin, petunidin, pelargonidin and malvidin glycosides. Different superscript letters within columns mean significant differences (*p* < 0.05).
